# Household Income Is Associated with the p53 Mutation Frequency in Human Breast Tumors

**DOI:** 10.1371/journal.pone.0057361

**Published:** 2013-03-01

**Authors:** Adrienne M. Starks, Damali N. Martin, Tiffany H. Dorsey, Brenda J. Boersma, Tiffany A. Wallace, Stefan Ambs

**Affiliations:** 1 Laboratory of Human Carcinogenesis, Center for Cancer Research, National Cancer Institute, National Institutes of Health, Bethesda, Maryland, United States of America; 2 Epidemiology and Genetics Research Program, Division of Cancer Control and Population Sciences, National Cancer Institute, National Institutes of Health, Bethesda, Maryland, United States of America; University of Illinois at Chicago, United States Of America

## Abstract

**Background:**

A study from Scotland reported that the p53 mutation frequency in breast tumors is associated with socio-economic deprivation.

**Methods:**

We analyzed the association of the tumor p53 mutational status with tumor characteristics, education, and self-reported annual household income (HI) among 173 breast cancer patients from the greater Baltimore area, United States.

**Results:**

p53 mutational frequency was significantly associated with HI. Patients with < $15,000 HI had the highest p53 mutation frequency (21%), followed by the income group between $15,000 and $60,000 (18%), while those above $60,000 HI had the fewest mutations (5%). When dichotomized at $60,000, 26 out of 135 patients in the low income category had acquired a p53 mutation, while only 2 out of 38 with a high income carried a mutation (*P* < 0.05). In the adjusted logistic regression analysis with 3 income categories (trend test), the association between HI and p53 mutational status was independent of tumor characteristics, age, race/ethnicity, tobacco smoking and body mass. Further analyses revealed that HI may impact the p53 mutational frequency preferentially in patients who develop an estrogen receptor (ER)-negative disease. Within this group, 42% of the low income patients (< $15,000 HI) carried a mutation, followed by the middle income group (21%), while those above $60,000 HI did not carry mutations (*P*_trend_ < 0.05).

**Conclusions:**

HI is associated with the p53 mutational frequency in patients who develop an ER-negative disease. Furthermore, high income patients may acquire fewer p53 mutations than other patients, suggesting that lifetime exposures associated with socio-economic status may impact breast cancer biology.

## Introduction

Breast cancer incidence and mortality rates show large differences among population groups within the United States (US) and between more and less developed countries worldwide [1]–[3]. Results from cancer epidemiology indicate that differences in reproductive history and exposures to certain lifestyle factors largely explain geographic and race/ethnic variations in the breast cancer incidence [4]. In contrast, population differences in mortality are thought to be caused by inadequate and delayed access to health care and by differences in disease presentation at diagnosis [5]–[10]. Recently, it has been argued that additional, yet unrecognized differences in tumor biology may exist that account for some the observed race/ethnic differences in disease survival in the US [11], [12].

It has been well established that a patient’s socioeconomic status (SES) is associated with breast cancer survival and may influence tumor characteristics like the mutational and estrogen receptor (ER) status of tumors. For example, women from a low SES background were found to be more likely to develop an ER-negative disease than those from a high SES background [13], [14]. Other studies described race/ethnic differences in the p53 tumor suppressor status of breast cancer patients in the US [15], [16], suggesting environmental and SES-related influences on tumor biology that lead to these differences. This hypothesis is consistent with the literature showing that the tumor p53 mutational status has an environmental signature and can sometimes be traced back to well defined exposures [17].

A study of 246 breast cancer patients in Scotland recently reported that the p53 mutational frequency in breast tumors is associated with socio-economic deprivation [18]. Women with the highest deprivation scores in the area of their residence were found to acquire p53 mutations more frequently than those living in other areas. This finding links socio-economic deprivation to a poor outcome phenotype because the presence of a p53 mutation in breast tumors confers decreased disease-free and overall survival [19], [20]. While intriguing, observations like these will need further validation in other patient groups. Thus, we tested the hypothesis that annual household income (HI), as a measure of SES, is associated with the p53 mutation frequency in a cohort of US breast cancer patients. Our study found that HI was associated with the p53 mutation frequency in breast tumors, and high income patients may acquire fewer p53 mutations than other patients. Moreover, our data revealed that HI may impact the p53 mutational frequency preferentially in patients who develop an ER-negative disease.

## Results

We previously established a well characterized cohort of 143 African-American and 105 European-American breast cancer patients with information on the tumor p53 mutational status and survival follow-up [21]. This patient population was recruited in the greater Baltimore area in Maryland, US, and is representative of an inner city, low income community with a large minority population. Many of the patients were from an impoverished background and 47 (27%) of them reported annual incomes less than $15,000. The high proportion of African-American patients explains the elevated frequency of high grade and ER-negative tumors in this patient population, which is representative of African-American breast cancer patients [4], [7], [22]. Self-reported HI was available for 173 of the 248 patients (70%). Comparing the patients with HI information (n  =  173) versus those without this information (n  =  75), there was no difference with respect to self-reported race/ethnicity (*P*  =  0.78), tumor ER status (*P*  =  1.0), node status (*P*  =  0.77), or body mass index (*P*  =  0.74), but patients with missing income information tended to be older (57.7 versus 54.0 mean age; *P*  =  0.05) and their tumors tended to have a higher grade (58% versus 46%; *P*  =  0.14) and a higher p53 mutation frequency (26.7% versus 16.2%; *P*  =  0.08).

To evaluate the association between tumor p53 status and selected patient and tumor characteristics, patients were stratified into no/yes mutation carriers and the association with tumor and patient characteristics was assessed (**Table 1**). The tumor p53 status was significantly associated with HI, node status, tumor ER status, number of tumor-infiltrating macrophages, and reached the *P*  =  0.05 significance level with tumor grade. Tobacco consumption in pack years tended to be higher in patients without p53 mutations while African-American patients were more likely to acquire a p53 mutation (20.4%) than European-American patients (10.7%), albeit this relationship was not statistically significant (*P*  =  0.1). An increased p53 mutation frequency in ER-negative and high grade tumors has been observed by others [23], [24], consistent with our findings. Somewhat unexpected was the inverse relationship between p53 mutations and disease node status in our study cohort, whereas other studies either did not find an association between the two variables [23], [24] or observed a positive relationship [18]. There was no association between p53 mutation frequency and age at diagnosis, education, or a patient’s body mass index (BMI). Patients with less than $15,000 HI had the highest p53 mutation frequency (10/47; 21%), followed by the income group between $15,000 and $60,000 (16/88; 18%), while those above $60,000 HI had the fewest mutations (2/38; 5%) [trend test: *P*  =  0.057 using logistic regression with income coded 0,1,2]. In the multivariable logistic regression analysis with 3 income categories, HI was significantly associated with the p53 mutation frequency after adjustment for node status, tumor ER status, tumor grade, and race/ethnicity [odds ratio (OR)  =  0.42, 95% CI: 0.18 to 0.97 for acquiring a tumor p53 mutation with increasing HI]. This association remained significant when tobacco consumption and additional demographic variables (age, education, BMI) were added to the model (**Table 2**). In contrast to the tumor p53 mutational status, we did not observe an independent association between HI and either tumor p53 protein expression or the tumor ER status. Aberrant accumulation of nuclear p53 protein is commonly associated with the presence of a p53 mutation although the prognostic significance of nuclear p53 expression in breast tumors has been questioned [19], [20]. HI and aberrant nuclear p53 accumulation in the breast tumors were inversely related in this study, but in contrast to the relationship between HI and the tumor p53 mutational status, this association was not significant in the multivariable analysis (**Table 2**). Finally, we explored whether the tumor p53 mutational status is associated with race/ethnicity in the adjusted logistic regression analysis because African-American patients tended to acquire a p53 mutation more commonly than European-American patients (**Table 1**). In the adjusted models, as shown in **Table 2**, no relationship between race/ethnicity and the tumor p53 mutational status remained (OR  =  1.03, 95% CI: 0.43 to 2.45, with adjustments for household income, tumor grade, and ER and node status; OR  =  1.05, 95% CI: 0.43 to 2.56, with an additional adjustment for smoking; OR = 1.11, 95% CI: 0.34 to 3.58, with further adjustments for age, education, and BMI). Thus, race/ethnicity was not independently associated with the tumor p53 mutation frequency, whereas household income was.

**Table 1:** Association of patient characteristics with tumor p53 mutational status.

	All Cases (n = 173)	p53 mutation No (n = 145)	p53 mutation Yes (n = 28)	*P* value* t-test
Age at diagnosis (mean ± SD; n = 173)	54.0 ± 13.3	54.0 ± 13.2	53.6 ± 14.4	0.89
Body mass index (mean ± SD; n = 168)**	28.9 ± 8.4	28.9 ± 8.7	29.1 ± 6.4	0.93
Tumor-associated macrophages (CD68) (mean ± SD; n = 172)**	97.5 ± 58.6	92.9 ± 57.3	120.8 ± 60.6	0.021
Smoking in pack years [mean (range); n = 164]**	9.8 (0–112)	10.3 (0–112)	7.6 (0–111)	0.08***
	N**	N (%)**	N (%)**	Fisher’s exact test
Race/ethnicity				0.10
African-American	98	78 (80)	20 (20)	
European-American	75	67 (89)	8 (11)	
Household income				0.045
≤ $60k/year	135	109 (81)	26 (19)	
> $60k/year	38	36 (95)	2 (5)	
Education**				1.0
No High school degree	39	33 (85)	6 (15)	
High school degree or more	132	110 (83)	22 (17)	
Stage at diagnosis (TNM) **				1.0
≤ Stage II	131	108 (82)	23 (18)	
Stage III/IV	25	21 (84)	4 (16)	
Node status**				0.009
Negative	99	77 (78)	22 (22)	
Positive	61	57 (93)	4 (7)	
Tumor grade**				0.050
Low (1 & 2)	81	72 (89)	9 (11)	
High (3)	70	53 (76)	17 (24)	
Estrogen receptor				0.034
Negative	71	54 (76)	17 (24)	
Positive	102	91 (89)	11 (11)	
p53 IHC				0.001
Negative	119	108 (91)	11 (9)	
Positive	54	37 (69)	17 (31)	

* *P* value comparing patient characteristics by tumor p53 mutational status

** Cases with missing information are not included

*** Mann-Whitney rank sum test

Annual household income, race/ethnicity, and education are self-reported. Tumor-associated macrophages were counted as CD68-positive cells. Pack years: (packs smoked per day) x (years as a smoker). BMI  =  kg/m^2^; SD  =  standard deviation, IHC  =  immunohistochemistry.

**Table 2:** Relationship between tumor p53 status and annual household income in the adjusted analysis.

	Adjusted logistic regression** OR (95% CI) (n = 143)	OR (95% CI) additionally adjusted for smoking (n = 136)	OR (95% CI) further adjusted for age, education, and BMI (n = 131)
Odds of acquiring a p53 mutation with increasing household income*	1	1	1
	**0.42 (0.18 to 0.97)**	**0.4 (0.17 to 0.94)**	**0.32 (0.10 to 0.99)**
Odds of acquiring a p53 IHC-positive tumor with increasing household income*			
	1	1	1
	**0.57 (0.30 to 1.08)**	**0.63 (0.33 to 1.21)**	**0.88 (0.40 to 1.97)**

OR  =  odds ratio; CI  =  confidence interval; IHC  =  immunohistochemistry

* Trend test. Shown is the OR for the stepwise increase in household income (reference: low income). Income coded as 0 (< $15,000), 1 ($15,000 to $60,000), and 2 (> $60,000); adjustments: smoking (pack years), age, and body mass index (BMI) were used as continuous data; other covariates were dichotomized for the analysis, as shown in Table 1

** adjusted for race/ethnicity, node status, tumor estrogen receptor status, and tumor grade

Because p53 mutations occur most commonly in the ER-negative disease and women from a low SES background were found to be more likely to develop an ER-negative disease than those from a high SES background, we performed an additional analysis of the association between HI and the p53 mutation frequency after stratification of the patients by tumor ER status. This analysis revealed that HI may impact the p53 mutational frequency preferentially in patients who develop the ER-negative disease, but not in ER-positive tumors (**Table 3**). Within this group of ER-negative patients, 42% of the lowest income patients carried a mutation, followed by the middle income group (21%), while those in the high income group did not carry any mutations (*P_trend_*  =  0.044).

**Table 3:** Association of household income with a mutant p53 tumor status by estrogen receptor status.

	All tumors		ER-positive tumors		ER-negative tumors	
**Household Income**	Wt p53	Mutant p53	Wt p53	Mutant p53	Wt p53	Mutant p53
**Low income < $15K**	37 (79%)	10 (21%)	26 (93%)	2 (7%)	11 (58%)	8 (42%)
**Middle income $15 to $60K**	72 (82%)	16 (18%)	38 (84%)	7 (16%)	34 (79%)	9 (21%)
**High Income > $60K**	36 (95%)	2 (5%)	27 (93%)	2 (7%)	9 (100%)	0 (0%)
***P*** **_trend_ (Fisher’s exact test)**	0.09		0.55		**0.044**	

**Figure 1:**
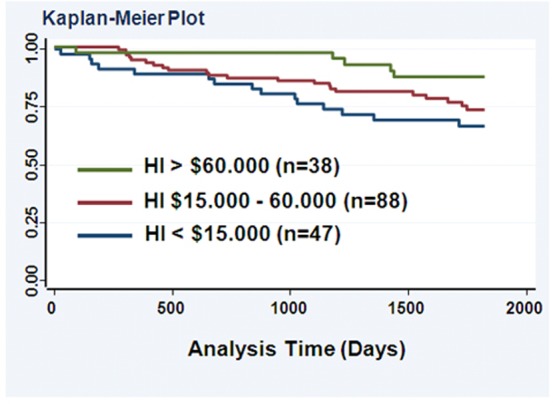
Kaplan-Meier survival curves for 5-year breast cancer-specific survival by annual household income (HI) of the patients. Log-rank test: *P* < 0.05. Within this follow-up period, 38 of the 173 patients (22%) died from breast cancer.

In an exploratory approach, we also examined the association of HI and the tumor p53 status with disease-specific survival using Cox regression modeling. Both HI (hazard ratio (HR)  =  0.64, 95% CI: 0.44 to 0.94 for dying from breast cancer with increasing HI) and the tumor p53 mutation status (HR  =  1.66, 95% CI: 1.02 to 2.7 for a p53 mutation carrier vs. non-carrier) were significantly associated with survival in the univariable analysis, but not education (HR  =  0.76, 95% CI: 0.45 to 1.26). **Figure 1** shows a Kaplan-Meier plot of the relationship between HI and breast cancer-specific survival. In an analysis that included both HI and tumor p53 mutation status as covariates, only HI was a significant predictor of survival. The inclusion of other covariates to the model that were associated with survival in the univariable analysis (age, TNM stage, ER status in addition to HI and p53 mutation status) yielded a borderline significant association for both HI (HR  =  0.62, 95% CI: 0.38 to 1.02 for dying from breast cancer with increasing HI) and tumor p53 status (HR  =  1.95, 95% CI: 0.93 to 4.1 for a p53 mutation carrier vs. non-carrier) with disease-specific survival, suggesting that these two variables are likely independent predictors of survival in larger studies. Additional analyses did not find that the two variables may affect survival through an interaction.

## Discussion

A mutation in the p53 tumor suppressor gene has been linked to poor disease outcome and therapy resistance in breast cancer [19], [20], [25], [26]. Thus, exposures that promote the development of p53 mutations will negatively affect therapy response and disease survival of breast cancer patients. Other studies observed that the p53 mutational spectrum in various human cancers can be linked to distinct carcinogen exposures like aflatoxin exposure and tobacco consumption, and unique mutational spectra have been observed in certain geographic area [17], [27], showing that the environment can influence tumor biology by affecting the tumor p53 mutational status.

Here, we report the finding that HI as a measure for individual-level SES is inversely associated with the p53 mutational frequency in breast cancer patients from the greater Baltimore area in the US, which corroborates previous observations from a study in Scotland showing that community-level SES had a similar effect [18]. Thus, socioeconomic factors may affect breast cancer biology by either increasing the risk of acquiring a tumor p53 mutation among those with a low income or decreasing it for those with a high income. We also discovered that HI was rather distinctively associated with the p53 mutational frequency in patients who develop an ER-negative disease, but not in those with the ER-positive disease (mostly luminal A & B tumors). This novel observation may at least be partly explained by the close relationship between the occurrence of a p53 mutation and the development of basal-like and triple-negative breast tumors [28], [29]. Thus, exposures associated with SES may specifically impact the breast tumor biology and development of ER-negative breast cancer subtypes by selecting for mutant p53 tumors, consistent with several epidemiologic studies that linked a low SES to an increased risk of developing ER-negative tumors [13], [14].

Our observation that high income patients acquired significantly fewer p53 mutations in their primary tumors than other patients contrasts with the findings in the Scottish study where differences were most obvious when breast cancer patients living in the most deprived areas were compared with those living in other areas. The Scottish study would suggest that harmful exposures in the most deprived areas cause an increased p53 mutation frequency whereas our study hints to a protective mechanism or absence of the harmful exposures amongst those with a high SES. The difference in observation may relate to differences in the studied populations, or to the fact that we used questionnaire data to describe SES while the Scottish study relied on area deprivation scores as a measure for SES. Nevertheless, the findings from the two studies are generally consistent because the observed direction of how SES influences the risk of developing a p53 mutant breast tumor was the same in these studies. The Scottish report also observed a positive interaction between deprivation and p53 mutations on survival, which indicated a potentially synergistic relationship between the two factors in increasing the risk for disease recurrence and death. While we observed that both HI and a tumor p53 mutation were associated with survival in US patients, we did not observe this interaction in our analyses. We interpret our finding with caution because it could either indicate that an interaction between household income and mutant p53 on survival did not exist in the US cohort or that our study was underpowered to detect this interaction. Nevertheless, the two studies are very consistent in their general finding and support the hypothesis that SES, either measured by area deprivation score or as household income, modifies the risk of acquiring a p53 mutation in breast cancer patients, and that both factors, socioeconomic deprivation and a p53 mutation, are associated with poor survival.

Currently, we do not know the mechanism by which socioeconomic deprivation may increase the risk of acquiring a p53 mutation in breast tissue. It is possible that certain environmental exposures associated with socio-economic deprivation impact breast tumor biology. For example, hormone replacement therapy, lack of physical activity, and increased alcohol consumption are recognized breast cancer risk factors [30], [31]. Exposure to these risk factors may vary between population groups [32]. An association between alcohol consumption and p53 mutation frequency in breast tumors of pre-menopausal women has been observed in one study [33] but the mechanism by which alcohol may cause p53 mutations in unknown. Some of these risk factors could indirectly affect the p53 mutation frequency by altering sex hormone metabolism and by increasing estradiol metabolite-induced mutagenesis [34]. One recent publication revealed a relationship between low early-life social class and increased pro-inflammatory signaling in healthy volunteers [35], which is particularly interesting since inflammation is a cancer risk factor and the expression of inflammation-induced enzymes like inducible nitric oxide synthase and activation-induced cytidine deaminase has been associated with an increased p53 mutational frequency [36]–[39]. Other studies have linked environmental stress exposures to increased disease aggressiveness in breast cancer [40]–[42]. Psychological stressors that are more common in deprived communities have been shown to have long-lasting effects on immunity and inflammation pathways [43], [44]; they may interact with environmental pollutants to increase the rate of p53 mutations in affected individuals and breast cancer patients. Nontheless, future research is needed to further delineate the mechanisms linking socio-economic deprivation to tumor p53 mutations.

Our study design has strengths and limitations that should be discussed. In contrast to the Scottish study, which used area deprivation scores as a surrogate for a patient’s SES, we had access to self-reported income and education data which more accurately represent SES of a patient. One limitation in our study is the relatively small sample size of the patient population and the fact that a subset of the patient population did not provide HI data. Because only 28 patients in the study presented with a p53 mutation, some of the multivariable logistic regression estimates could be unreliable. However, it should be noted that the estimates of the relationship between HI (income coded 0,1,2) and the tumor p53 mutational status were relatively stable using logistic regression models starting with the unadjusted analysis (OR  =  0.55, 95% CI: 0.3 to 1.02) and then applying the models shown in 2. Moreover, the observed association between HI and the p53 mutation frequency remained statistically significant in the multivariable analysis and our observations are consistent with a previously published report. A second limitation relates to the patient cohort. This cohort is representative of an inner city, low income community with a large minority population. These patients developed ER-negative and high grade disease more commonly than expected for women in the US in general and may not be representative for US and European patient populations. However, the association between HI and the tumor p53 mutation frequency remained statistically significant in the multivariable analysis after adjustments for tumor ER status and grade. Furthermore, one may see it as a strength to find that SES was associated with the tumor p53 status in two different patient populations, one from Baltimore with a large African-American population and one from Scotland with a mostly white population. Thirdly, we did not have adequate exposure data for some breast cancer risk factors like alcohol consumption and hormone replacement therapy and could not examine their relationship with the tumor p53 status in this patient population. Lastly, it is possible that our analysis missed TP53 mutations, although we used three methods (single-stranded conformation polymorphism, the GeneChip p53 assay, and direct sequencing) to detect TP53 mutations in microdissected tumor samples.

In conclusion, our study corroborates a previous observation from a study in Scotland that p53 mutant tumors are more common in breast cancer patients from low income, socially deprived communities than in patients from high SES communities, indicating that lifetime exposures associated with a woman’s SES may impact breast tumor biology. The current study extends the previous observations by showing that SES may preferentially impact the biology of the ER-negative disease. These findings are significant in light of the ongoing discussions why women from disadvantaged communities tend to have poorer survival than women from high income area. In addition to the existing disparities in access to care, SES may impact tumor biology, leading to a poor outcome phenotype with mutant p53 that more commonly affects low income patients than high income patients.

## Materials and Methods

### Patient recruitment and survey data

Unselected breast cancer patients at all disease stages were recruited between February 15, 1993, and August 27, 2003, into a biomarker study under a NCI contract (Resource Collection and Evaluation of Human Tissues and Cells from Donors with an Epidemiology Profile), as described previously [21]. They were recruited at the University of Maryland Medical Center, the Baltimore Veterans Affairs Medical Center, Union Memorial Hospital, Mercy Medical Center, and the Sinai Hospital in Baltimore. They were identified through surgery lists and enrolled into the study prior to surgery. Resected tumor tissue (for p53 mutational analysis) was obtained from 248 patients who had pathologically confirmed breast cancer, were of African-American or European-American descent by self-report, and had been diagnosed with breast cancer within the last 6 months before recruitment, and had, by self-report, no previous history of breast cancer. Patients completed an interviewer-administered questionnaire that evaluated socio-economic variables as part of a larger survey. Self-reported HI was available for 173 (70%) of them, and information on education for 171 (69%). Eighteen patients, or their doctor, refused that the questionnaire is administered and 24 patients could not be interviewed while others refused to complete the income section or were not able to report household income. Combined annual household income before taxes and deductions was collected as either unknown, under $15,000, between $15,000 and $60,000, and above $60,000. Education information was collected as highest grade or level of schooling and how many years of school were completed. Self-reported race/ethnicity was collected as Black (not of Hispanic origin) for African-Americans and White (not of Hispanic origin) for European-Americans. Clinical and pathological information including tumor hormone receptor expression was obtained from medical records and pathology reports and HER2 expression was evaluated by immunohistochemistry as described by us previously [45]. Disease staging was performed according to the tumor–node–metastasis (TNM) classification system of the American Joint Committee on Cancer/ the Union Internationale Contre le Cancer (AJCC/UICC). The Nottingham system was used to determine tumor grade.

### Ethics Statement

All patients signed a consent form. The collection of tumor specimens, survey data, and clinical and pathological information was approved by the University of Maryland Institutional Review Board for the participating institutions (UMD protocol #0298229). IRB approval of this protocol was then obtained at all participating institutions. The research was also reviewed and approved by the NIH Office of Human Subjects Research (OHSR #2248).

### *TP53* mutational analysis and immunohistochemistry

Tumors were screened by single-stranded conformation polymorphism analysis for the presence of somatic p53 mutations, as previously described [46], [47]. p53 exons 5−8 were amplified by polymerase chain reaction (PCR) from microdissected, paraffin-embedded tumor tissue, as described [48]. PCR products were denaturated into single-stranded DNA and loaded on a Gene Gel Excel gel (Amersham Pharmacia Biotech, Piscataway, NJ). The single-stranded DNA was separated by electrophoresis by use of the GenePhor System (Amersham Pharmacia Biotech, Piscataway, NJ) and visualized by DNA silver staining. When an aberrant DNA band pattern was detected, the PCR product was sequenced to determine whether a mutation was present. Most tumors were also screened for mutations in p53 exons 2–11 with the GeneChip p53 assay (Affymetrix, Santa Clara, CA). This protocol has been previously validated [33], [49]. Predicted mutations were scored as described [50]. Nuclear p53 expression was determined immunohistochemically with a 1:100 diluted monoclonal DO-7 antibody (DakoCytomation, Carpinteria, CA; recognizes the N-terminus of the p53 protein); p53 expression was scored positive if >10% of the tumor cells expressed nuclear p53.

### Statistical analysis

Data analysis was performed using Stata/SE 9.0 (Stata Corp, College Station, TX) statistical software package. All statistical tests were two-sided, and an association was considered statistically significant with *P* < 0.05. The t-test, Fisher’s exact test and the Mann-Whitney rank sum test, and multivariable logistic regression, were used for statistical analyses and to calculate odds ratios, respectively. Survival was determined for the period from the date of hospital admission to the date of the last search for death entries in the Social Security Index (date of search: December 31^st^, 2006). We obtained information (National Death Index, death certificates) on the causes of death for the deceased patients and censored all patients whose causes of death were not related to breast cancer. For logistic and Cox regression analyses, patients were stratified into no/yes mutation carriers and household income was stratified into three categories (< $15,000, between $15,000 and $60,000, > $60,000). Education was dichotomized into no high school degree and having a high school degree or above.

## References

[1] Kamangar F, Dores GM, Anderson WF (2006) Patterns of cancer incidence, mortality, and prevalence across five continents: defining priorities to reduce cancer disparities in different geographic regions of the world. J Clin Oncol 24: 2137–2150. 16682732 10.1200/JCO.2005.05.2308

[2] Forouzanfar MH, Foreman KJ, Delossantos AM, Lozano R, Lopez AD, et al (2011) Breast and cervical cancer in 187 countries between 1980 and 2010: a systematic analysis. Lancet 378: 1461–1484. 21924486 10.1016/S0140-6736(11)61351-2

[3] Desantis C, Siegel R, Bandi P, Jemal A (2011) Breast cancer statistics, 2011. CA Cancer J Clin 61: 409–418. 21969133 10.3322/caac.20134

[4] Chlebowski RT, Chen Z, Anderson GL, Rohan T, Aragaki A, et al (2005) Ethnicity and breast cancer: factors influencing differences in incidence and outcome. J Natl Cancer Inst 97: 439–448. 15770008 10.1093/jnci/dji064

[5] Tammemagi CM, Nerenz D, Neslund-Dudas C, Feldkamp C, Nathanson D (2005) Comorbidity and survival disparities among black and white patients with breast cancer. JAMA 294: 1765–1772. 16219879 10.1001/jama.294.14.1765

[6] Hershman D, McBride R, Jacobson JS, Lamerato L, Roberts K, et al (2005) Racial disparities in treatment and survival among women with early-stage breast cancer. J Clin Oncol 23: 6639–6646. 16170171 10.1200/JCO.2005.12.633

[7] Carey LA, Perou CM, Livasy CA, Dressler LG, Cowan D, et al (2006) Race, breast cancer subtypes, and survival in the Carolina Breast Cancer Study. JAMA 295: 2492–2502. 16757721 10.1001/jama.295.21.2492

[8] Curtis E, Quale C, Haggstrom D, Smith-Bindman R (2008) Racial and ethnic differences in breast cancer survival: how much is explained by screening, tumor severity, biology, treatment, comorbidities, and demographics? Cancer 112: 171–180. 18040998 10.1002/cncr.23131 PMC2674622

[9] van Ravesteyn NT, Schechter CB, Near AM, Heijnsdijk EA, Stoto MA, et al (2011) Race-specific impact of natural history, mammography screening, and adjuvant treatment on breast cancer mortality rates in the United States. Cancer Epidemiol Biomarkers Prev 20: 112–122. 21119071 10.1158/1055-9965.EPI-10-0944 PMC3075821

[10] Wallace TA, Martin DN, Ambs S (2011) Interactions among genes, tumor biology and the environment in cancer health disparities: examining the evidence on a national and global scale. Carcinogenesis 32: 1107–1121. 21464040 10.1093/carcin/bgr066 PMC3149201

[11] Newman LA, Griffith KA, Jatoi I, Simon MS, Crowe JP, et al (2006) Meta-analysis of survival in African American and white American patients with breast cancer: ethnicity compared with socioeconomic status. J Clin Oncol 24: 1342–1349. 16549828 10.1200/JCO.2005.03.3472

[12] Albain KS, Unger JM, Crowley JJ, Coltman CA Jr, Hershman DL (2009) Racial disparities in cancer survival among randomized clinical trials patients of the Southwest Oncology Group. J Natl Cancer Inst 101: 984–992. 19584328 10.1093/jnci/djp175 PMC2724852

[13] Gordon NH (1995) Association of education and income with estrogen receptor status in primary breast cancer. Am J Epidemiol 142: 796–803. 7572955 10.1093/oxfordjournals.aje.a117718

[14] Thomson CS, Hole DJ, Twelves CJ, Brewster DH, Black RJ (2001) Prognostic factors in women with breast cancer: distribution by socioeconomic status and effect on differences in survival. J Epidemiol Community Health 55: 308–315. 11297648 10.1136/jech.55.5.308 PMC1731899

[15] Jones BA, Kasl SV, Howe CL, Lachman M, Dubrow R, et al (2004) African-American/White differences in breast carcinoma: p53 alterations and other tumor characteristics. Cancer 101: 1293–1301. 15368321 10.1002/cncr.20500

[16] Martin DN, Boersma BJ, Yi M, Reimers M, Howe TM, et al (2009) Differences in the Tumor Microenvironment between African-American and European-American Breast Cancer Patients. PLoS ONE 4: e4531. 19225562 10.1371/journal.pone.0004531 PMC2638012

[17] Greenblatt MS, Bennett WP, Hollstein M, Harris CC (1994) Mutations in the p53 tumor suppressor gene: clues to cancer etiology and molecular pathogenesis. Cancer Res 54: 4855–4878. 8069852

[18] Baker L, Quinlan PR, Patten N, Ashfield A, Birse-Stewart-Bell LJ, et al (2010) p53 mutation, deprivation and poor prognosis in primary breast cancer. Br J Cancer 102: 719–726. 20104224 10.1038/sj.bjc.6605540 PMC2837559

[19] Pharoah PD, Day NE, Caldas C (1999) Somatic mutations in the p53 gene and prognosis in breast cancer: a meta-analysis. Br J Cancer 80: 1968–1973. 10471047 10.1038/sj.bjc.6690628 PMC2363143

[20] Soussi T, Beroud C (2001) Assessing TP53 status in human tumours to evaluate clinical outcome. Nature Rev Cancer 1: 233–240. 11902578 10.1038/35106009

[21] Boersma BJ, Howe TM, Goodman JE, Yfantis HG, Lee DH, et al (2006) Association of breast cancer outcome with status of p53 and MDM2 SNP309. J Natl Cancer Inst 98: 911–919. 16818855 10.1093/jnci/djj245

[22] Stead LA, Lash TL, Sobieraj JE, Chi DD, Westrup JL, et al (2009) Triple-negative breast cancers are increased in black women regardless of age or body mass index. Breast Cancer Res 11: R18. 19320967 10.1186/bcr2242 PMC2688946

[23] Thor AD, Moore DHII, Edgerton SM, Kawasaki ES, Reihsaus E, et al (1992) Accumulation of p53 tumor suppressor gene protein: an independent marker of prognosis in breast cancers. J Natl Cancer Inst 84: 845–855. 1317462 10.1093/jnci/84.11.845

[24] Sasa M, Kondo K, Komaki K, Morimoto T, Monden Y (1994) p53 alteration correlates with negative ER, negative PgR, and high histologic grade in breast cancer. J Surg Oncol 56: 46–50. 8176940 10.1002/jso.2930560110

[25] Aas T, Borresen AL, Geisler S, Smith-Sorensen B, Johnsen H, et al (1996) Specific P53 mutations are associated with de novo resistance to doxorubicin in breast cancer patients. Nat Med 2: 811–814. 8673929 10.1038/nm0796-811

[26] Olivier M, Langerod A, Carrieri P, Bergh J, Klaar S, et al (2006) The clinical value of somatic TP53 gene mutations in 1,794 patients with breast cancer. Clin Cancer Res 12: 1157–1167. 16489069 10.1158/1078-0432.CCR-05-1029

[27] Hussain SP, Harris CC (1999) p53 mutation spectrum and load: the generation of hypotheses linking the exposure of endogenous or exogenous carcinogens to human cancer. Mutat Res 428: 23–32. 10517975 10.1016/s1383-5742(99)00028-9

[28] Calza S, Hall P, Auer G, Bjohle J, Klaar S, et al (2006) Intrinsic molecular signature of breast cancer in a population-based cohort of 412 patients. Breast Cancer Res 8: R34. 16846532 10.1186/bcr1517 PMC1779468

[29] The Human Cancer Genome Network (2012) Comprehensive molecular portraits of human breast tumours. Nature 490: 61–70. 23000897 10.1038/nature11412 PMC3465532

[30] Hamajima N, Hirose K, Tajima K, Rohan T, Calle EE, et al (2002) Alcohol, tobacco and breast cancer--collaborative reanalysis of individual data from 53 epidemiological studies, including 58,515 women with breast cancer and 95,067 women without the disease. Br J Cancer 87: 1234–1245. 12439712 10.1038/sj.bjc.6600596 PMC2562507

[31] Narod SA (2011) Hormone replacement therapy and the risk of breast cancer. Nat Rev Clin Oncol 8: 669–676. 21808267 10.1038/nrclinonc.2011.110

[32] Bernstein L, Teal CR, Joslyn S, Wilson J (2003) Ethnicity-related variation in breast cancer risk factors. Cancer 97: 222–229. 12491485 10.1002/cncr.11014

[33] Freudenheim JL, Bonner M, Krishnan S, Ambrosone CB, Graham S, et al (2004) Diet and alcohol consumption in relation to p53 mutations in breast tumors. Carcinogenesis 25: 931–939. 14742318 10.1093/carcin/bgh088

[34] Fernandez SV, Russo IH, Russo J (2006) Estradiol and its metabolites 4-hydroxyestradiol and 2-hydroxyestradiol induce mutations in human breast epithelial cells. Int J Cancer 118: 1862–1868. 16287077 10.1002/ijc.21590

[35] Miller GE, Chen E, Fok AK, Walker H, Lim A, et al (2009) Low early-life social class leaves a biological residue manifested by decreased glucocorticoid and increased proinflammatory signaling. Proc Natl Acad Sci U S A 106: 14716–14721. 19617551 10.1073/pnas.0902971106 PMC2732821

[36] Ambs S, Bennett WP, Merriam WG, Ogunfusika MO, Oser SM, et al (1999) Relationship between p53 mutations and inducible nitric oxide synthase expression in human colorectal cancer. J Natl Cancer Inst 91: 86–88. 9890175 10.1093/jnci/91.1.86

[37] Vaninetti NM, Geldenhuys L, Porter GA, Risch H, Hainaut P, et al (2008) Inducible nitric oxide synthase, nitrotyrosine and p53 mutations in the molecular pathogenesis of Barrett's esophagus and esophageal adenocarcinoma. Mol Carcinog 47: 275–285. 17849424 10.1002/mc.20382

[38] Glynn SA, Boersma BJ, Dorsey TH, Yi M, Yfantis HG, et al (2010) Increased NOS2 predicts poor survival in estrogen receptor-negative breast cancer patients. J Clin Invest 120: 3843–3854. 20978357 10.1172/JCI42059 PMC2964971

[39] Igarashi H, Hashimoto J, Tomita T, Yoshikawa H, Ishihara K (2010) TP53 mutations coincide with the ectopic expression of activation-induced cytidine deaminase in the fibroblast-like synoviocytes derived from a fraction of patients with rheumatoid arthritis. Clin Exp Immunol 161: 71–80. 20491788 10.1111/j.1365-2249.2010.04163.x PMC2940151

[40] Hermes GL, Delgado B, Tretiakova M, Cavigelli SA, Krausz T, et al (2009) Social isolation dysregulates endocrine and behavioral stress while increasing malignant burden of spontaneous mammary tumors. Proc Natl Acad Sci U S A 106: 22393–22398. 20018726 10.1073/pnas.0910753106 PMC2799783

[41] Sloan EK, Priceman SJ, Cox BF, Yu S, Pimentel MA, et al (2010) The sympathetic nervous system induces a metastatic switch in primary breast cancer. Cancer Res 70: 7042–7052. 20823155 10.1158/0008-5472.CAN-10-0522 PMC2940980

[42] Pan D, Kocherginsky M, Conzen SD (2011) Activation of the Glucocorticoid Receptor Is Associated with Poor Prognosis in Estrogen Receptor-Negative Breast Cancer. Cancer Res 71: 6360–70. 21868756 10.1158/0008-5472.CAN-11-0362 PMC3514452

[43] Antoni MH, Lutgendorf SK, Cole SW, Dhabhar FS, Sephton SE, et al (2006) The influence of bio-behavioural factors on tumour biology: pathways and mechanisms. Nat Rev Cancer 6: 240–248. 16498446 10.1038/nrc1820 PMC3146042

[44] Miller GE, Chen E, Parker KJ (2011) Psychological stress in childhood and susceptibility to the chronic diseases of aging: moving toward a model of behavioral and biological mechanisms. Psychol Bull 137: 959–997. 21787044 10.1037/a0024768 PMC3202072

[45] Glynn SA, Prueitt RL, Ridnour LA, Boersma BJ, Dorsey TM, et al (2010) COX-2 activation is associated with Akt phosphorylation and poor survival in ER-negative, HER2-positive breast cancer. BMC Cancer 10: 626. 21078168 10.1186/1471-2407-10-626 PMC2993681

[46] Piao CQ, Willey JC, Hei TK (1999) Alterations of p53 in tumorigenic human bronchial epithelial cells correlate with metastatic potential. Carcinogenesis 20: 1529–1533. 10426802 10.1093/carcin/20.8.1529

[47] Colomer A, Erill N, Verdu M, Roman R, Vidal A, et al (2003) Lack of p53 nuclear immunostaining is not indicative of absence of TP53 gene mutations in colorectal adenocarcinomas. Appl Immunohistochem Mol Morphol 11: 130–137. 12777996 10.1097/00129039-200306000-00007

[48] Coombs NJ, Gough AC, Primrose JN (1999) Optimisation of DNA and RNA extraction from archival formalin-fixed tissue. Nucleic Acids Res 27: e12. 10454649 10.1093/nar/27.16.e12 PMC148555

[49] Ahrendt SA, Halachmi S, Chow JT, Wu L, Halachmi N, et al (1999) Rapid p53 sequence analysis in primary lung cancer using an oligonucleotide probe array. Proc Natl Acad Sci U S A 96: 7382–7387. 10377423 10.1073/pnas.96.13.7382 PMC22094

[50] Ahrendt SA, Hu Y, Buta M, McDermott MP, Benoit N, et al (2003) p53 mutations and survival in stage I non-small-cell lung cancer: results of a prospective study. J Natl Cancer Inst 95: 961–970. 12837832 10.1093/jnci/95.13.961

